# The complete mitochondrial genome of the endangered freshwater fish *Odontobutis obscurus* from Korea

**DOI:** 10.1080/23802359.2022.2054736

**Published:** 2022-03-28

**Authors:** Kang-Rae Kim, Yeong-Ho Kwak, Mu-Sung Sung, Gun Young Lee, Yong Hwi Kim, In-Chul Bang

**Affiliations:** a Department of Life Science & Biotechnology, Soonchunhyang University, Asan, Republic of Korea; b Inland Fisheries Research Institute, National Institute of Fisheries Science, Gapyeong, Republic of Korea

**Keywords:** Complete mitochondrial genome, *Odontobutis obscurus*, Endangered fish, freshwater fish

## Abstract

We report the first complete mitochondrial genome of *Odontobutis obscurus*, which consists of 17,038 bp harboring 13 protein-coding genes, two ribosomal RNA genes, 22 transfer RNA genes, and a control region (D-loop). The overall base composition of the complete genome is A (30.63%), C (28.72%), T (25.70%), G (14.95%). The complete mitogenome of *Odontobutis obscurus*, most closely related to congeners in the Bayesian inference and maximum likelihood tree, provides a better understanding of the phylogeny of the genus *Odontobutis*.

The dark sleeper *Odontobutis obscurus* Temminck & Schlegel, 1845 is a freshwater fish native to China, Japan, and Korea. Although the dark sleeper is not on the IUCN Red List category, it was evaluated as Critically Endangered (CR) on the National Red List in Korea because it inhabits only Geojedo Island, Korea. Therefore, in Korea, it has been protected as a Class I endangered wild animal designated by the Ministry of Environment (Ministry of Environment (ME) 2012).

Studies have investigated the distribution and habitat of *O*. *obscurus*, but no genetic studies have been published. Therefore, we report on the mitogenome of *O*. *obscurus* to enable genetic comparisons between China, Japan, and South Korea and present the data as a basis for species conservation.

A specimen of *O*. *obscurus* was collected from Sanyangcheon Stream, South Korea (34°49'N, 128°36'E) on 30 November 2011. This occurred before *O. obscurus* was designated an endangered species. Therefore, research ethics approval was waived according to Soonchunhyang University Institutional Animal Care and Use Committee (IACUC) regulations, and the author did not need permits for land access in the field study. The specimen was immediately placed in 99.9% ethanol and stored under voucher number SUC-26727 in the fish specimen room of Soonchunhyang University (voucher Storage: Soonchunhyang University; voucher number: SUC-26727; person in charge of the collection: KR Kim; email: kimkangrae9586@gmail.com). Genomic DNA was extracted from the caudal fin using the Genomic DNA Prep Kit (BioFact, Daejeon, South Korea) and stored at −80 °C. A DNA library was prepared to obtain the complete mitochondrial DNA sequence. DNA library preparation was conducted using the MGIEasy DNA Library Prep Kit (MGI Technology, Shenzhen, China) according to the manufacturer’s instructions, and sequencing was performed with 150 base pairs (bp) paired-ends using the MGISEQ-2000 platform (MGI Technology). The raw data obtained from the MGISEQ-2000 platform were assembled using Geneious 11.0.3 (https://www.geneious.com) and annotated with the assembled mitogenome sequences using the MITOS web server (Bernt et al. 2013). The complete mitogenome sequence of *O*. *obscurus* was deposited at the National Center for Biotechnology Information GenBank under accession number MW646297.

The mitochondrial genome of *O*. *obscurus* consists of 17,038 bp, harboring 13 protein-coding genes (PCGs), two ribosomal RNA (rRNA) genes, 22 transfer RNA (tRNA) genes, and a control region (D-loop). Two PCGs (CO1 and ND3) start with a GTG codon and the other 11 PCGs start with ATG. The overall base composition is A (30.63%), C (28.72%), T (25.70%), and G (14.95%), with a high AT content of 56.33%. The two rRNAs are 16S (1,675 bp) and 12S (951 bp).

**Figure 1.**
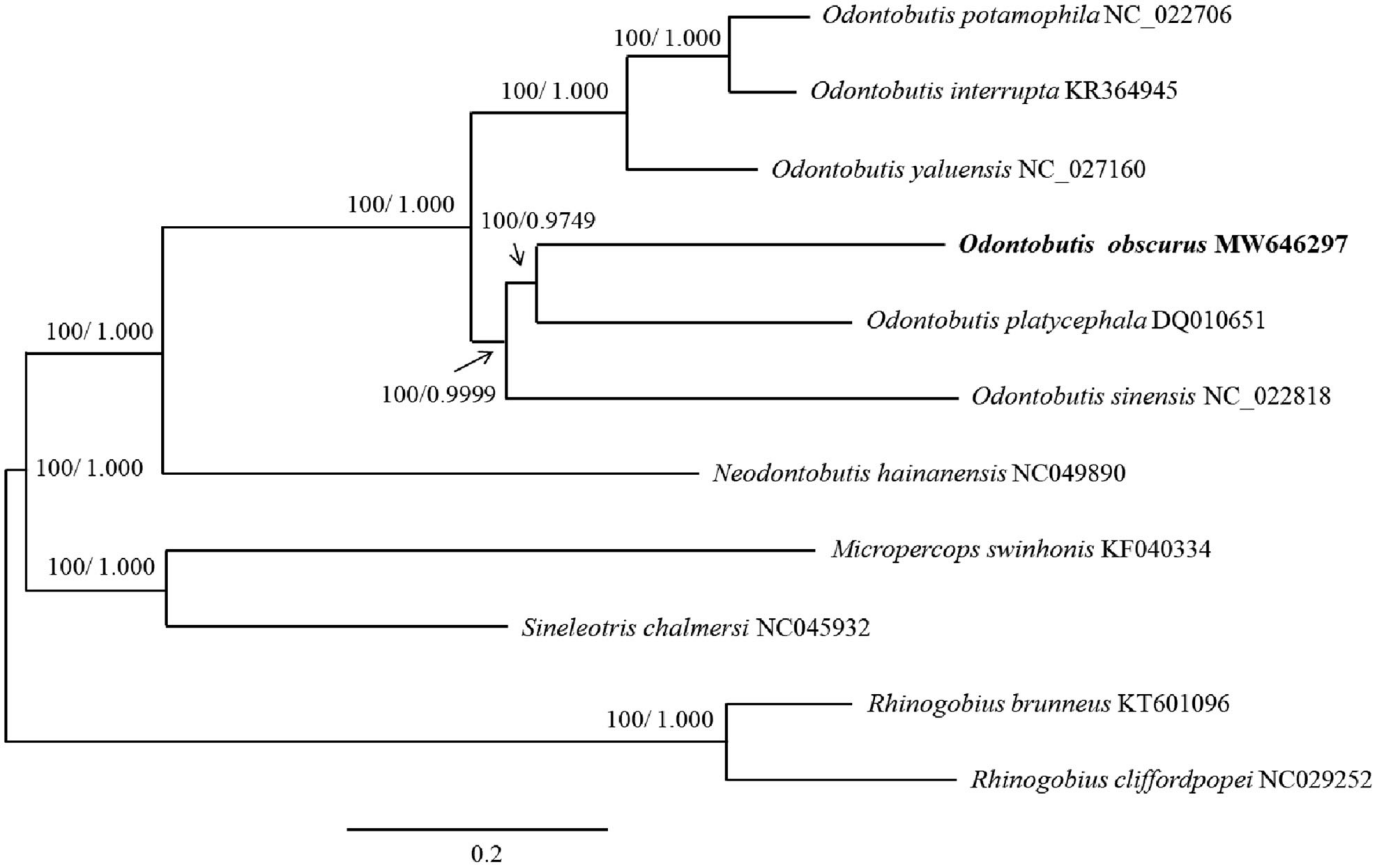
Phylogenetic tree constructed for the genus *Odontobutis*, with outgroup species, using the Bayesian inference and maximum likelihood methods based on 13 protein-coding genes. The numbers above the nodes represent the bootstrap support values and posterior probabilities of Bayesian inference for each branch.

The dataset was aligned using the ClustalW module of MEGA X and the TIM2 + I + G model was the best fit nucleotide substitution model as calculated by jModelTest 2.1.10 (Guindon and Gascuel 2003; Darriba et al. 2012). A phylogenetic tree was constructed based on 13 PCGs using the Bayesian inference (MrBayes 3.2.7) and maximum likelihood (PhyML 3.0) methods (Guindon et al. 2010; Ronquist et al. 2012), which grouped *O*. *obscurus* in the genus *Odontobutis* (Figure 1).

## Data Availability

The genome sequence data that support the findings of this study are openly available in GenBank of NCBI at (https://www.ncbi.nlm.nih.gov/) under the accession no. MW646297. The associated BioProject, SRA, and Bio-Sample numbers are PRJNA752481, SRX11659825 and SAMN20599215, respectively.
